# Functional connectivity changes in the thalamocortical network due to neck pain and the multiscale regulatory effects of acupuncture: a cross-scale multi-omics neuroimaging study

**DOI:** 10.3389/fnins.2026.1783910

**Published:** 2026-04-28

**Authors:** Zhen Gao, Jing Zhang, Jia-Hui Chang, Hai-Jun Wang, Cheng Xu, Lai-Xi Ji

**Affiliations:** 1 Experimental Management Center, Shanxi University of Traditional Chinese Medicine, Taiyuan, Shanxi, China; 2 Shanxi Key Laboratory of Jinpai Acupuncture, Taiyuan, China; 3 Acupuncture and Moxibustion Department II, Affiliated Hospital of Shanxi University of Traditional Chinese Medicine, Taiyuan, Shanxi, China; 4 Second Clinical Medical College, Shanxi University of Traditional Chinese Medicine, Taiyuan, Shanxi, China; 5 Department of Radiology, Shanxi Provincial People’s Hospital, Taiyuan, Shanxi, China

**Keywords:** acupuncture, functional magnetic resonance imaging, multi-scale mechanisms, neck pain, thalamus

## Abstract

**Background:**

Neck pain correlates with multiscale brain abnormalities, but cross-scale mechanisms of acupuncture analgesia are unclear. This study aimed to: (1) Explore differential modulation of thalamic functional networks by verum vs. sham acupuncture; (2) Examine associations between functional connectivity changes and micro gene expression to unravel its multiscale mechanisms.

**Methods:**

A total of 130 participants were initially enrolled, and 100 eligible neck pain patients were randomized 1:1 to the verum (*n* = 50) or sham (*n* = 50) acupuncture groups. Finally, 49 patients in each group were included for the final analysis due to one case of exclusion in each group, with treatment administered twice a week for 2 weeks. Visual Analog Scale (VAS), resting-state functional magnetic resonance imaging (fMRI), and Allen Human Brain Atlas (AHBA) transcriptome data were analyzed via Partial Least Squares (PLS) regression.

**Results:**

Both groups showed reduced post-treatment VAS (*p* < 0.001), with the verum group exhibiting a superior effect (*Z* = −6.877, *p* < 0.001). Neuroimaging revealed that verum acupuncture (VA) specifically induced significant decreases in functional connectivity (FC) between the right thalamus and left anterior cingulate cortex (*T* = −4.498) as well as between the right thalamus and right Rolandic operculum (*T* = −4.532, voxel-level *p* < 0.01, cluster-level *p* < 0.05), an effect absent in the sham acupuncture group (SA). Gene- FC association analysis indicated that PLS2 component explained 39.83% of FC variance (*P*_spin_: permutation test *p* < 0.05), with weight genes showing significant spatial correlation to connectivity changes (*r* = 0.445, *P*_spin_ = 0.0011). A total of 809 genes were enriched in the innate immune response and phosphorylation regulation pathways, whereas 1,222 genes were enriched in the GABA-ergic synapse and synaptic membrane-related pathways.

**Conclusion:**

VA relieves pain via modulating thalamus-anterior cingulate cortex networks, involving immune-inflammation and neural inhibition, providing first multi-scale validation integrating neuroimaging and transcriptomics.

**Clinical trial registration:**

This trial was registered with the International Traditional Medicine Clinical Trial Registry (registration number: ITMCTR2023000001) prior to participant enrollment.

## Introduction

Neck pain represents a highly prevalent and frequently encountered clinical condition associated with multiscale alterations in the brain. Neuroimaging studies have revealed macroscopic functional changes across various brain regions in neck pain patients. Concurrently, at the microscopic level, significant biochemical alterations occur within neurotransmitter systems and related molecular pathways (Karlsson et al., 2015). These multiscale brain abnormalities form a complex pathological network that underpins the persistence of neck pain symptoms, and thus therapeutic interventions for neck pain should not only target these macroscopic cerebral functional changes but also investigate and regulate the underlying microscopic neurochemical mechanisms driving these adaptive and pathological alterations.

As a classic non-pharmacological intervention with a long history of clinical application in pain management, acupuncture alleviates neck pain through mechanisms operating at multiple biological levels, which aligns with the intrinsic multiscale pathological characteristics of neck pain. Empirical evidence indicates its ability to modulate macroscopic neural networks, including enhanced FC between the thalamus and raphe nuclei, which correlates with clinical improvement in pain symptoms (Wang et al., 2024). Simultaneously, at the microscopic level, acupuncture regulates neurotransmitter receptor activity and associated molecular pathways to produce therapeutic effects (Wang et al., 2020; Wang et al., 2022). Nevertheless, existing research has not yet integrated macroscopic neuroimaging data with microscopic molecular profiling to comprehensively elucidate the cross-scale mechanisms underlying acupuncture efficacy for neck pain.

fMRI is a non-invasive neuroimaging technique that detects hemodynamic changes associated with brain activity, enabling investigation of brain function and its relationship with behavior. Using the thalamus as a seed region, Lv et al. demonstrated widespread reductions in effective connectivity between the thalamus and global brain regions in pain patients (Lv et al., 2024). Acupuncture may alleviate chronic pain by modulating trigeminospinothalamic-cortical circuitry (Mercer Lindsay et al., 2021), with the thalamus recognized as a critical brain region for acupuncture treatment of musculoskeletal pain (Ha et al., 2022). Given this evidence, enhancing thalamic function has emerged as a key therapeutic strategy for neck pain relief. However, whether acupuncture ameliorates pain symptoms through modulation of thalamic functional networks remains an unresolved question requiring further elucidation.

Therefore, this randomized controlled trial investigates the mechanistic role of thalamic functional networks in acupuncture treatment for neck pain. The study has dual objectives: first, to compare differences in thalamic resting-state FC before and after verum versus sham acupuncture interventions; second, to identify associations between gene expression profiles and acupuncture-induced resting-state FC changes through multimodal integration of neuroimaging and transcriptomic data. We hypothesize that: (1) VA, but not sham acupuncture, will alleviate neck pain symptoms and modulate thalamic functional networks in patients; (2) Acupuncture-induced alterations in FC will demonstrate significant spatial correlations with specific neurotransmitter-related gene expression patterns.

## Methods

### Study design

This single-center, randomized, sham-acupuncture controlled trial was conducted from March 2023 to May 2024. The protocol received ethical approval from the Institutional Review Board of Shanxi Hospital of Acupuncture and Moxibustion (Approval No. 2023–007) and was prospectively registered with the International Traditional Medicine Clinical Trial Registry (Registration ID: ITMCTR2023000001). All participants provided written informed consent prior to enrollment. The study adheres to the Consolidated Standards of Reporting Trials (CONSORT) guidelines.

The study duration was 3 weeks, comprising a 1-week baseline period followed by a 2-week acupuncture intervention phase with treatment sessions administered twice weekly. A total of 130 participants were initially enrolled, and 100 eligible neck pain patients were randomly allocated to two acupuncture groups in a 1:1 ratio (50 patients per group). All participants underwent MRI scans at both baseline and post-treatment timepoints.

### Patient selection

Participants were recruited from Shanxi University of Chinese Medicine and Shanxi Hospital of Acupuncture and Moxibustion. Diagnosis was confirmed by board-certified orthopedic physicians using the 2017 Clinical Practice Guidelines for Neck Pain (Orthopaedic Section, American Physical Therapy Association) incorporating the International Classification of Functioning, Disability and Health framework (Blanpied et al., 2017). Inclusion criteria comprised: (1) mechanical neck pain diagnosis; (2) VAS score >3 (0–10 range); (3) age 18–60 years; (4) right-handedness; (5) written informed consent. Exclusion criteria were: (1) pregnancy/lactation; (2) communication-impairing psychiatric/neurological disorders; (3) MRI contraindications (e.g., claustrophobia, metallic implants); (4) concurrent clinical trial participation.

### Sample size calculation

As an exploratory neuroimaging investigation, this study acknowledges the current lack of consensus regarding sample size calculation in functional MRI research. With reference to prior studies indicating that 15 participants per group represents the minimum feasible sample size for detecting neural effects (Hayasaka et al., 2007), and considering both clinical experience and preliminary findings in published literature, a total sample size of 100 patients (50 per group) was determined for MRI acquisition to ensure robust statistical power.

### Acupuncture interventions

Neck pain patients were randomized 1:1 to either VA or SA groups using computer-generated allocation sequences. Throughout the study, patients, outcome assessors, data collectors, and statisticians remained blinded to group assignment. All licensed acupuncturists involved in this trial had at least 3 years of experience in acupuncture and completed standardized clinical trial protocol training.

For patients in the VA group, pressure pain threshold (PPT) was measured using a digital algometer (Model FPX25). The site with the lowest PPT was identified as the acupuncture point. Based on previous studies, five body regions with the highest frequency of pain and the greatest acupoint sensitivity were selected for PPT detection (Sun et al., 2019). The probe of the algometer was applied perpendicular to the skin surface, and pressure was applied at a constant rate of 1 kg/cm^2^/s until the subject reported pain, at which point the measurement was stopped and the value was recorded. The four sites with the lowest PPT values were selected as the acupuncture points for intervention. Licensed acupuncturists performed either perpendicular or oblique needling (depth adjusted per anatomical site) followed by rotational manipulation (180° amplitude, 2 Hz frequency) for 1 min to elicit deqi sensation (characteristic needling sensation). Needles were retained for 30 min, with manual stimulation reapplied for 1 min at 10-min intervals using identical manipulation parameters.

For patients in the SA group, acupuncture was performed at non-acupoint locations. The needles were inserted at an oblique angle of 15° relative to the skin surface to a depth of 2–3 mm, and no manipulation was performed to elicit the deqi sensation. We selected sham points that had been previously validated and were confirmed not to elicit specific physiological effects (Sun et al., 2019; Melchart et al., 2005). Specifically, sham point 1 was located at the midpoint between the tip of the elbow and the axillae, while sham point 2 was positioned in the middle of the line connecting the insertion of the deltoid muscle (LI 14) and the acromion. The needles were retained for 30 min.

### Clinical outcome measurement

The primary outcome measure used in this study was the VAS score, a well-established scale for quantifying the severity of pain symptoms. Clinical assessments were conducted at two time points: baseline and after treatment.

### MRI acquisition

MRI scans were performed on all participants before treatment and after completion of either VA or SA. All MRI data were acquired using a 3.0 T MR scanner (Siemens 3 T Tim trio, Germany) at the Shanxi Provincial People’s Hospital, with the following specific parameters (Gao et al., 2025): 3D T1-weighted imaging using magnetization prepared rapid gradient echo: Repetition Time (TR) 1,900 ms, Echo Time (TE) 2.26 ms, Field of View (FOV) 256 × 256 mm^2^, Matrix 256 × 256. Resting-state fMRI scans were conducted using echo-planar imaging: TR 2,000 ms, TE 30 ms, FOV 240 × 240 mm^2^, Matrix 64 × 64, Flip Angle 90°, number of slices 31, slice thickness 5 mm, with a total of 240 time points acquired. Participants were instructed to keep their eyes closed and avoid any head movement during the scanning process.

### MRI data preprocessing

The preprocessing of resting-state fMRI data was performed using the Data Processing and Analysis of Brain Imaging (DPABI 4.3) and Statistical Parametric Mapping 12 (SPM12) software packages, which were run on MATLAB 2018a. The preprocessing pipeline included the following steps: DARTEL-based temporal slice timing correction (voxel size 3 × 3 × 3 mm^3^), head motion correction, segmentation, normalization to MNI space, Gaussian smoothing with a 6 mm full-width at half-maximum, detrending, regression of covariates, and exclusion of subjects with head motion exceeding 2.5 mm.

### Seed-based FC analyses

For FC analysis, the thalamus was selected as the seed region. Based on the MNI coordinates defined in the literature, spherical seed regions with a radius of 3 mm were constructed bilaterally: left thalamus (*x* = −14, *y* = −24, *z* = 2) and right thalamus (*x* = 14, *y* = −24, *z* = 2). Pearson correlation analysis was performed between the mean time series of these seed regions and the time series of all voxels in the whole brain, calculating the Pearson correlation coefficient (r). These correlation coefficients were then normalized using *Fisher’s Z* transformation to obtain the FC maps for each seed region. Two-sample *t*-tests were conducted on the FC values of the subjects using the SPM12 software. All analyses were corrected using the Gaussian Random Field (GRF) method, with a voxel-level threshold of *p* < 0.01 and a cluster-level threshold of *p* < 0.05.

### Gene expressional profiles obtainment and preprocessing

Gene expression profiles were extracted from six postmortem brain tissues obtained from the AHBA database.^1^ Gene expression analysis was limited to the left hemisphere, as only two donors in the AHBA database had right hemisphere data available, consistent with previous analyses (Estevez-Fraga et al., 2023). The following preprocessing steps were applied to the AHBA transcriptome data: (1) Probe-to-gene annotation was verified using the Re-Annotator toolkit; (2) Probes were filtered to retain those expressed above background levels in at least 50% of the samples; (3) Representative probes were selected by calculating the correlation between microarray datasets and RNA sequencing measurements, retaining probes with a correlation coefficient greater than 0.2, and selecting the most representative probe based on the strongest correlation with RNA sequencing data; (4) Tissue samples were assigned to the AAL116 brain atlas; (5) A scale-robust sigmoid normalization was applied to effectively minimize expression differences between genes within samples and between samples for each gene; (6) Gene sets were filtered based on differential stability. Since all donors had left hemisphere data available, only samples from the left hemisphere were retained. As a result, a transcriptional expression matrix was obtained, comprising 116 brain regions and 15,633 corresponding genes.

### Correlation analysis between transcription and regional changes before and after treatment

The PLS component signifies a linear combination of gene expression values, and generally, the first or second component (PLS1 or PLS2) is often recognized as the most effective low-dimensional representation for interpreting the covariance of the high-dimensional data matrix (Yao et al., 2024). To determine if the covariance explained between the FC t-statistic maps and transcriptomic scores in the PLS component was greater than what would be expected by chance, we performed a spatial autocorrelation analysis using a permutation test with 10,000 iterations. Bootstrapping was used to estimate the variability of PLS2 for each gene. The Z-score for each gene was calculated as the ratio of its weight to its bootstrap standard error, and genes were ranked according to their contribution to PLS2 (Li et al., 2021). Genes with a false discovery rate (FDR) of 5%, either for positive or negative PLS2, represented the FC gene list associated with regional changes.

### Enrichment pathways associated with changes in FC

We selected PLS2 as the optimal component, as it explained the highest degree of variance. Based on the PLS2 weights, important genes were divided into two distinct lists, termed PLS2 + genes (*FDR-corrected p* < 0.05) and PLS2- genes (*FDR-corrected p* < 0.05). To further elucidate the functional characteristics of these significant PLS +/− genes, we conducted enrichment analysis using Metascape.^2^ This analysis encompassed Gene Ontology (GO). A significance threshold of 5% was set, with corrections applied using FDR. To explore the link between the PLS2 gene lists and the polygenic risk of acupuncture analgesia, a protein–protein interaction (PPI) network was constructed for the shared genes.

## Results

### Baseline characteristics of the enrolled patients

**Figure 1:**
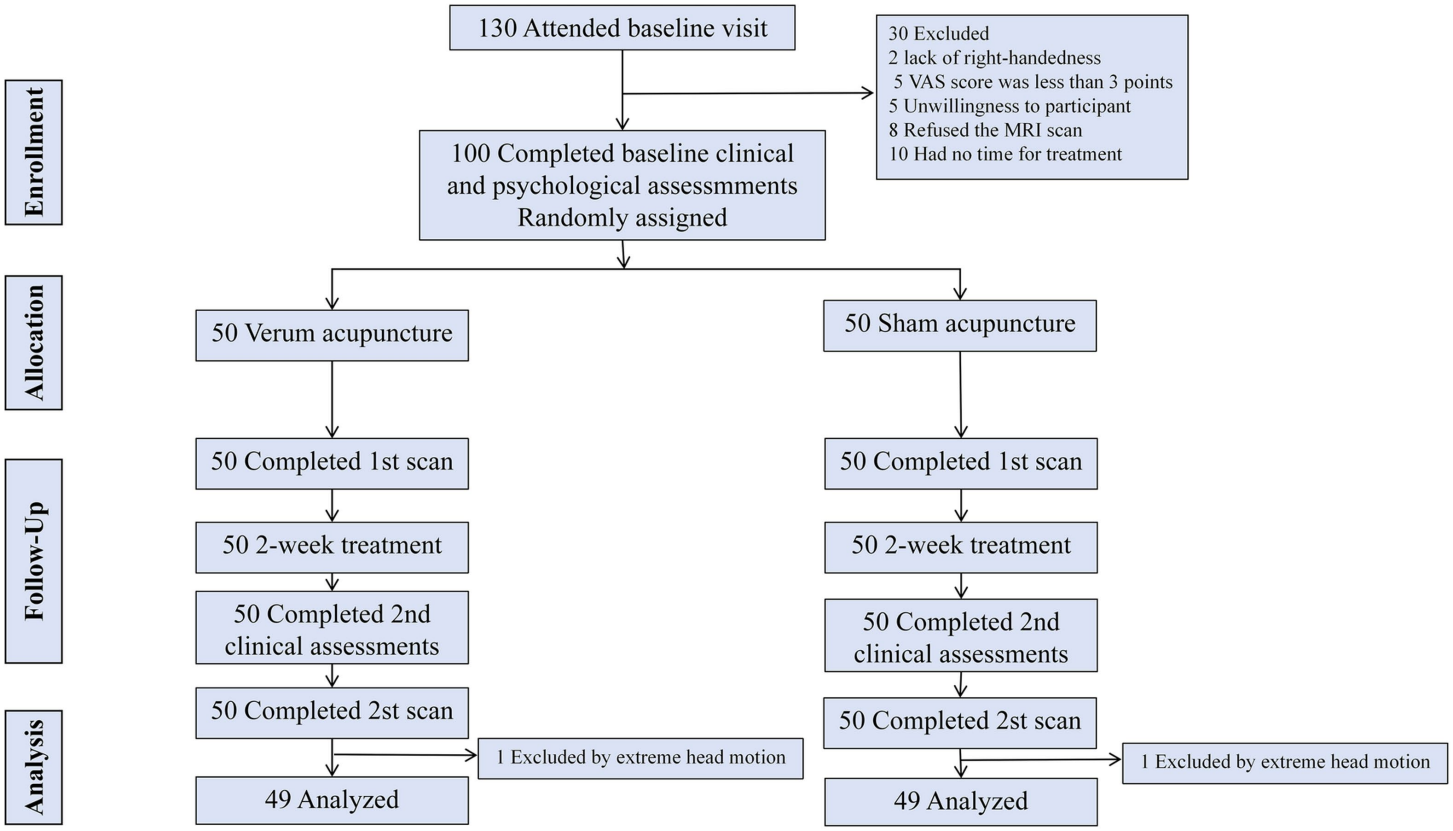
CONSORT flow diagram of the study. CONSORT, Consolidated Standards of Reporting Trials.

A total of 130 participants were initially enrolled in this study (Figure 1), among which 100 eligible neck pain patients were randomized to the VA (*n* = 50) and SA (*n* = 50) groups. One patient from the VA group and one from the SA group were excluded from the neuroimaging analysis due to excessive head movement. Ultimately, 49 patients in the VA group and 49 in the SA group were included in the final statistical and neuroimaging analysis. There were no statistically significant differences between the two groups in demographic characteristics, including gender, age, height, weight, duration of illness, and VAS scores (Table 1).

**Table 1:** Baseline characteristics of patients in both groups.

Clinical features	Verum acupuncture (*n* = 49)	Sham acupuncture (*n* = 49)	Statistical value	*P*-value
Age [median (P25, P75)]	24(21, 26)	24(22, 25)	*Z* = −0.064	0.949
Female(n)	36	29	χ^2^ = 2.239	0.199
Male(n)	13	20		
Disease course [medium (P25, P75)]	23 (14, 34.5)	25 (16, 30)	*Z* = −0.367	0.714
Height [medium (P25, P75)]	162(158, 168.5)	166(159.5, 172)	*Z* = −1.452	0.146
Weight [medium (P25, P75)]	55(53, 62)	58(50, 69.5)	*Z* = −0.865	0.387
VAS scores (mean ± SD)	5.74 ± 1.02	5.47 ± 0.93	*t* = 0.744	0.459

### Clinical outcomes

Within-group analysis showed that, compared to baseline, the VAS scores were significantly reduced after 2 weeks of VA group (*t* = 13.856, *p* < 0.001) and after 2 weeks of SA group (*t* = 6.197, *p* < 0.001). Between-group analysis revealed a significant difference in the change of VAS scores between the VA group (*Δ* = 3.00 ± 1.52) and the SA group (*Δ* = 1.16 ± 1.31) (*Z* = −6.877, *p* < 0.001) (Table 2).

**Table 2:** Pre-and post-treatment VAS differences between the two groups (paired *t*-test).

Group	Pre-treatment	Post-treatment	Statistical value	*P*-value
Verum acupuncture *n* = 49 (mean ± SD)	5.74 ± 1.02	2.74 ± 1.25	*t* = 13.856	<0.001
Sham acupuncture *n* = 49 (mean ± SD)	5.47 ± 0.93	4.31 ± 1.13	*t* = 6.197	<0.001

### Acupuncture effects on the thalamus functional network

**Figure 2:**
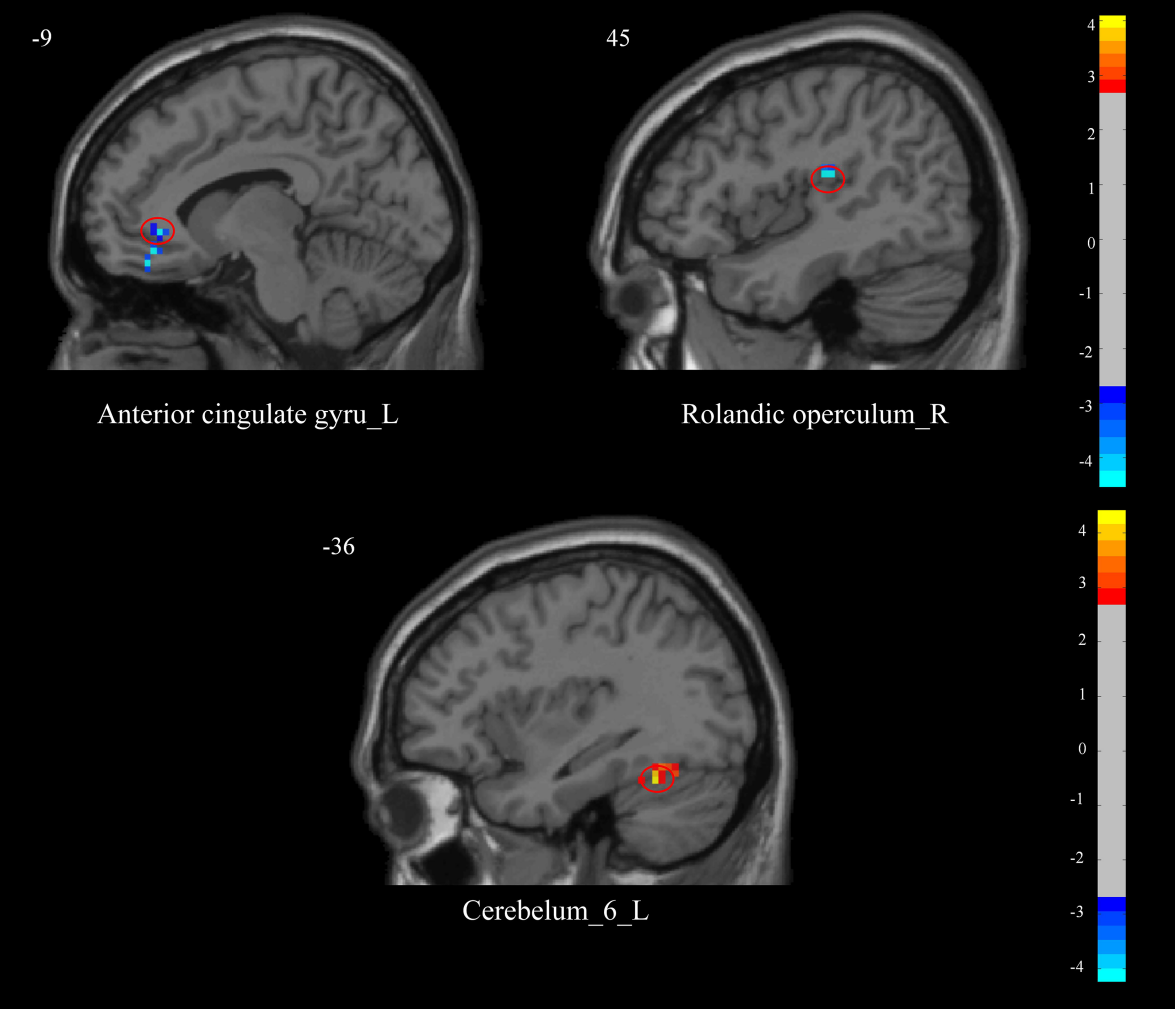
Differences in brain regions of functional connectivity before and after treatment in two groups of neck pain patients. The blue color indicates brain regions where functional connectivity significantly decreased in patients after treatment compared to before treatment; the red color indicates brain regions where functional connectivity significantly increased in patients after treatment compared to before treatment. For rs-fMRI-based functional connectivity analysis, two-sample *t*-tests were applied to participants’ FC values (SPM12), with GRF correction (voxel-level *p* < 0.01, cluster-level *p* < 0.05). L, left; R, right.

Using the right thalamus as the seed point, in the VA group, post-treatment compared to pre-treatment, there was a decrease in FC values between the right thalamus and the left Anterior Cingulate Gyrus (ACC) and the right Rolandic Operculum (voxel-level *p* < 0.01, cluster-level *p* < 0.05). In the SA group, post-treatment compared to pre-treatment, there was an increase in FC values between the right thalamus and the left Cerebellum_6 (*T* = 4.362, voxel-level *p* < 0.01, cluster-level *p* < 0.05) (Figure 2 and Table 3).

**Table 3:** Differences in brain regions of functional connectivity before and after treatment in two groups.

Brain region	Hemisphere	Cluster size	MNI peak coordinates			*T*-value
			X	Y	Z	
Anterior cingulate gyrus	Left	79	−9	36	−3	−4.498
Rolandic operculum	Right	34	45	−21	18	−4.532
Cerebellum_6	Left	28	−36	−51	−24	4.362

Based on GRF correction (voxel-level *P* < 0.01, cluster-level *P* < 0.05). GRF, Gaussian Random Field.

Using the left thalamus as the seed point, there were no significant differences in FC values before and after treatment in either the VA group or the SA group.

### Transcriptional patterns associated with regional changes before and after treatment

**Figure 3:**
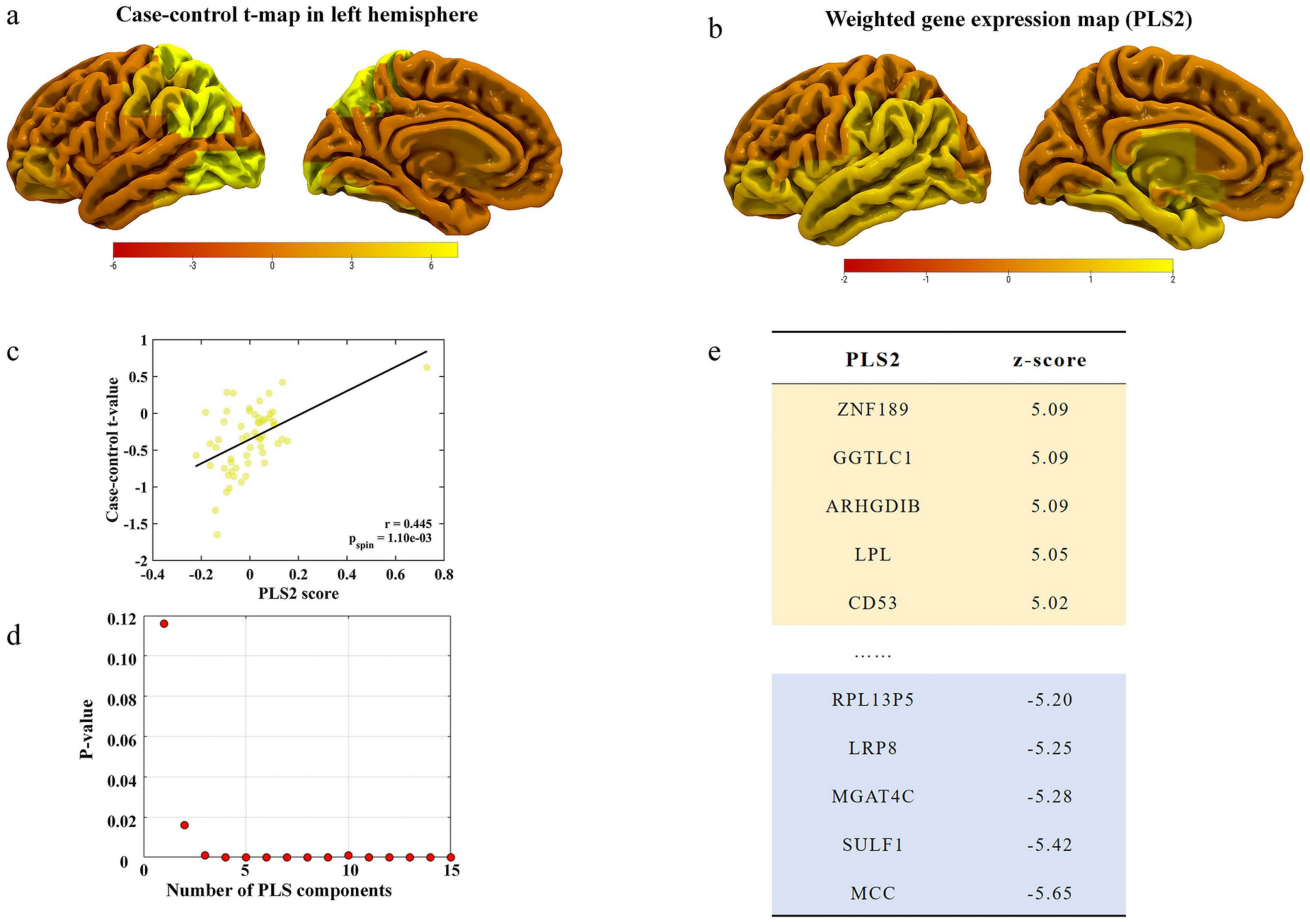
The gene expression profile associated with functional connectivity differences. **(a)** Changes in functional connectivity in the left hemisphere. **(b)** A weighted gene expression map of regional PLS2 scores in the left hemisphere. **(c)** A scatterplot of regional PLS2 scores and regional changes in functional connectivity (*Pearson’s r* = 0.445, *P*_spin_ = 0.001). **(d)** *p*-values for each component of PLS. **(e)** Ranked PLS2 loadings.

Based on the brain tissue transcription matrix (58 regions × 15,633 genes), we performed PLS regression to reveal the differences in functional activity in the left hemisphere brain regions associated with gene expression before and after treatment in the VA group (Figure 3a). Consequently, PLS1 and PLS2 effectively explained 13.26 and 39.83% of the variance in macroscopic FC differences before and after treatment in the VA group, respectively, significantly exceeding the random expectation (*P*_spin_ < 0.05). The distribution of the weighted gene expression profiles for PLS2 reflected a front-to-back gene expression gradient (Figure 3b), which was interpreted as the regional variation in the human cortical transcriptional architecture.

Furthermore, the PLS2 scores showed a significant spatial positive correlation with the case–control *t*-value maps before and after treatment in the VA group, indicating that genes weighted by PLS2 were co-overexpressed in regions with increased FC before and after treatment (*Pearson’s r* = 0.445, *P*_spin_ = 0.0011) (Figure 3c). Through univariate one-sample Z-tests, we successfully identified the PLS2-weighted genes and further categorized them into two gene lists, comprising 809 PLS2 + genes and 1,222 PLS2- genes (*Z* < −2.724, *FDR-corrected p* < 0.05) (Figures 3d,e and Supplementary Data 1).

### Enrichment pathways associated with changes in FC

**Figure 4:**
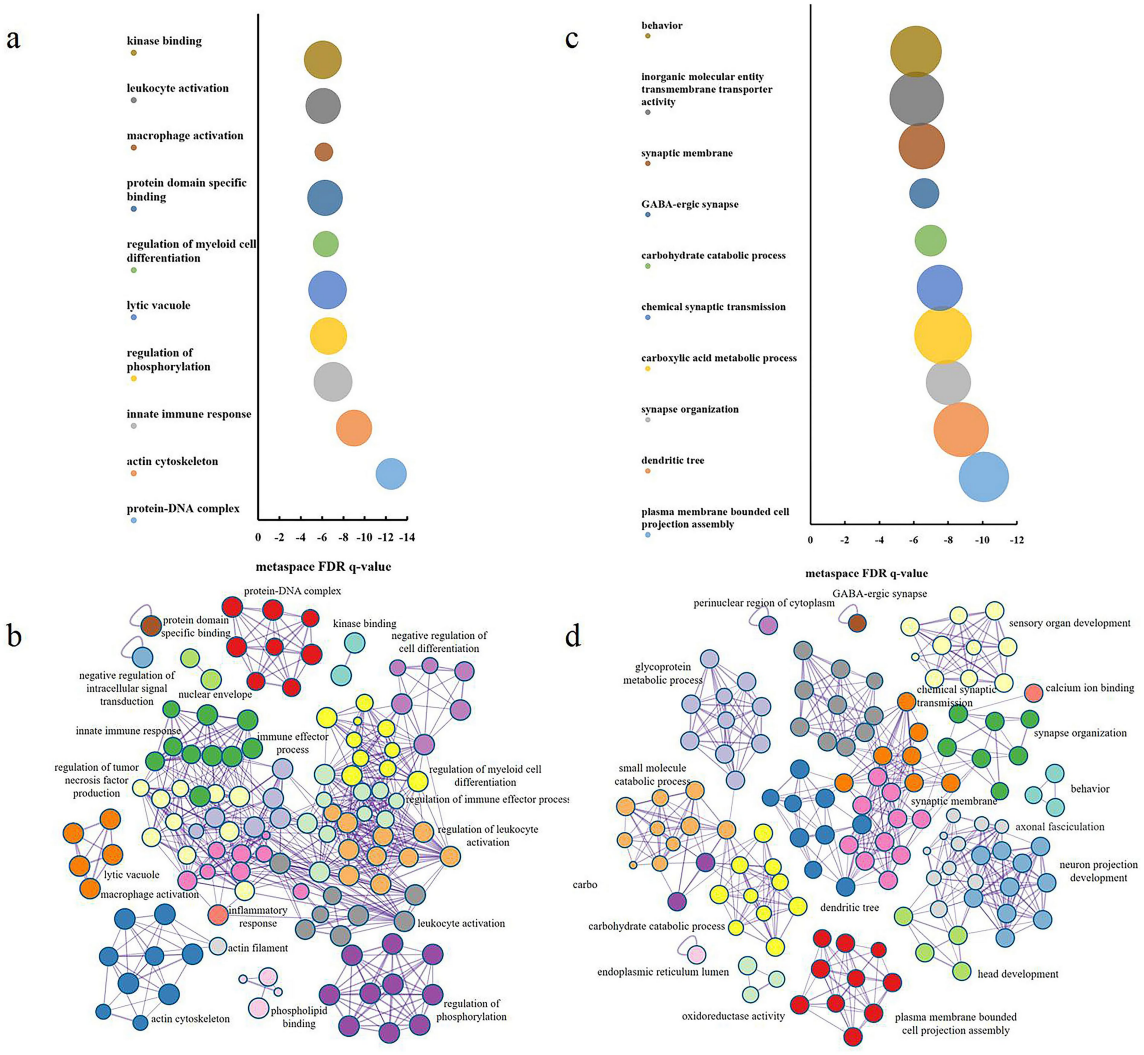
Enrichment analysis. **(a)** PLS2 + gene ontology terms (*FDR-corrected p* < 0.05). The circle size represents the number of genes associated with each term. **(c)** PLS2-gene ontology terms (*FDR-corrected p* < 0.05). **(b,d)** Metascape enrichment network visualization showing intra-cluster and inter-cluster term similarities. Each term is represented by a circle node, with size proportional to the number of input genes and color indicating cluster identity (i.e., nodes of the same color belong to the same cluster). Functional enrichment analysis was performed using Metascape (https://metascape.org/gp/index.html#/main/step1), a tool for Gene Ontology (GO) enrichment analysis and visualization. The statistical significance threshold was set at a false discovery rate (FDR) < 0.05.

We used Metascape to perform GO enrichment analysis on the PLS2- and PLS2 + gene lists. After correcting the enrichment terms (*FDR-corrected p* < 0.05) and discarding scattered enrichment clusters, the most significant Cellular Components for PLS2 + included “protein-DNA complex” and “actin cytoskeleton”; the most significant Biological Processes included “innate immune response,” “regulation of phosphorylation,” “macrophage activation,” “leukocyte activation” and “inflammatory response”; and the most significant Molecular Functions included “protein domain specific binding” and “kinase binding” (Figures 4a,b).

The results for PLS2- showed that the most significant Cellular Components included “GABA-ergic synapse” and “synaptic membrane”; the most significant Biological Processes included “carboxylic acid metabolic process” and “chemical synaptic transmission”; and the most significant Molecular Functions were primarily involved in “inorganic molecular entity transmembrane transporter activity” and “calcium ion binding” (Figures 4c,d).

## Discussion

To our knowledge, this is the first study to combine functional MRI and transcriptomic data to elucidate the central, cross-scale regulatory mechanisms of acupuncture analgesia. The results showed that: (1) there were aberrant functional connections in the thalamus and anterior cingulate cortex before and after VA treatment, and (2) the improvement in pain symptoms was closely related to aberrations in gene expression at the micro-level, which “drive” the abnormal changes in thalamic FC networks at the macro-level, thereby promoting pain recovery.

### Macro-level: the thalamus-anterior cingulate complex network is involved in the acupuncture analgesia process

The thalamus and ACC constitute a critical neural circuit for acupuncture analgesia. Anatomically, dense bidirectional fiber connections exist between these regions (Delevich et al., 2015), providing the structural foundation for nociceptive signal transmission and modulation. Functionally, the thalamus serves as a sensory relay hub that integrates ascending nociceptive signals from the periphery with non-noxious afferent inputs from acupuncture stimulation. In contrast, the ACC operates as a higher-order processing center that encodes the affective-motivational dimension of pain (e.g., unpleasantness, fear) and mediates cognitive appraisal (Kobayashi, 2012). Crucially, the ACC initiates potent descending inhibitory pathways that suppress pain transmission at the spinal cord level (Kwon et al., 2014). Consistent with our findings, studies confirm that FC between prefrontal cortices (including the ACC) and the thalamus represents a key neurobiological target for acupuncture in chronic pain management (Ong et al., 2019). Collectively, this integrated thalamocingulate circuitry enables multidimensional pain regulation, establishing itself as the central hub mediating acupuncture’s therapeutic effects (Wang et al., 2021).

Previous neuroimaging studies have provided compelling evidence. Research indicates that acupuncture significantly improves symptoms in pain patients and is associated with improved spontaneous neural activity in the thalamus and the ACC (Ha et al., 2022; Li et al., 2024). In patients with chronic pain, such as low back pain, the restoration of abnormal connectivity patterns in the ACC after acupuncture is significantly correlated with clinical pain relief (Li et al., 2014). Notably, the characteristic FC pattern between the thalamus and the ACC can effectively distinguish VA from sham acupuncture, highlighting its central role in mediating the specific effects of acupuncture (Kong et al., 2023). Furthermore, the cumulative analgesic effect of acupuncture is closely related to adaptive changes in neural responses within the thalamus (and its associated circuitry) (Li et al., 2014). These findings not only elucidate the neural mechanisms underlying acupuncture analgesia but also provide important targets for understanding pain modulation and optimizing treatment.

Furthermore, our study observed a significant decrease in FC of thalamic-related brain regions after acupuncture, which contrasts sharply with findings in painful diabetic peripheral neuropathy, where FC between the left thalamus and primary somatosensory cortex, as well as between the left thalamus and insular cortex, was found to increase significantly following cessation of pain treatment (Sloan et al., 2024). This discrepancy arises essentially from dual differences in pathological pain types and intervention strategies. As an exogenous active intervention, acupuncture modulates the central nociceptive conduction pathway, reversing the abnormally elevated FC of the thalamocortical pain processing network to normal physiological levels in patients with mechanical neck pain. Notably, mechanical neck pain is characterized by abnormal affective pain processing pathways mediated by the thalamus-anterior cingulate cortex, which differs fundamentally from the somatosensory integration pathways (thalamus-S1/thalamus-insula) involved in diabetic peripheral neuropathy. This divergence in core regulatory targets further leads to the opposite trends in FC changes. These results clearly demonstrate that the remodeling pattern of the central pain processing network is specific to both intervention approaches and pathological pain types, and our findings further enrich the understanding of acupuncture-mediated regulation of the thalamocortical network in non-neuropathic pain, providing neuroimaging evidence for precise acupuncture intervention in distinct pain types.

Notably, a significant increase in FC between the right thalamus and the left Cerebellum_6 was observed in the SA group, and this characteristic change reflects a non-specific central regulatory mechanism that is distinctly different from that of the VA group. The cerebellum is a core brain region in the central nervous system that regulates somatic movement (Block and Bastian, 2012), while the thalamus acts as a crucial relay station for sensory and motor signals, capable of effectively integrating and transmitting peripheral somatic sensory signals and central motor regulatory signals. Shallow needling stimulation at non-acupoints in the SA group did not trigger specific pain regulatory pathways, but instead induced a non-specific adaptive response in the central motor regulatory system. This response indirectly alleviates pain-induced motor limitations and muscle tension in the neck by modulating motor-related neural circuits, which also serves as an important neural basis for the partial analgesic effect of sham acupuncture. This finding further corroborates the core advantage of VA in achieving specific analgesia by targeted inhibition of pain-related pathological circuits such as the thalamus-anterior cingulate cortex, and also provides a potential biomarker for distinguishing the specific therapeutic effects of acupuncture from non-specific placebo effects at the imaging level.

### Micro-level: gene expression dysregulation “drives” the recovery of macroscopic brain FC

Pain is a complex phenomenon involving changes in multiple cellular components, molecular functions, and biological processes. We hypothesize that the macroscopic regulation of the brain by acupuncture treatment in pain patients might be driven by micro-level factors (such as gene expression and neurotransmitter distribution). The differences between PLS2 + and PLS2- provide a crucial perspective for us to gain a deeper understanding of the mechanisms of acupuncture.

Our study found that PLS2 + genes were significantly enriched in biological processes including innate immune response, macrophage/leukocyte activation and inflammatory response. This enrichment signature directly explains the correlation between the central immune-inflammatory pathway and abnormal FC in the thalamus-ACC, and also serves as a crucial molecular pathway for acupuncture to regulate this circuit. The persistence of neck pain is closely associated with the abnormal activation of central immune-inflammatory responses: peripheral inflammatory mediators induced by cervical soft tissue injury can cross the blood–brain barrier to activate microglia in the thalamus and ACC, leading to excessive release of pro-inflammatory factors in these brain regions (Long et al., 2023; Blaszczyk et al., 2018). This in turn enhances the excitability of nociceptive neurons, ultimately manifesting as abnormally elevated FC between the two regions. As a key switch in cellular signal transduction, phosphorylation directly regulates the release of inflammatory factors and the activity of immune cells. As classic intracellular signaling pathways that regulate phosphorylation and immune inflammation, MAPK and NF-κB mediate phosphorylation modifications, which represent the core molecular mechanism underlying the reduction of inflammatory factor release (Liu et al., 2025; Cheng et al., 2025). Based on existing research evidence, it is hypothesized that acupuncture may regulate such classic phosphorylation-related inflammatory signaling pathways to reduce the release of inflammatory factors within the thalamus-anterior cingulate cortex circuit, thereby lowering the excitability of the pain-related neural network and reversing the abnormally elevated functional connectivity between the thalamus and the anterior cingulate cortex. This molecular regulatory process establishes a direct link between the central immune-inflammatory pathway and the remodeling of FC in the thalamus-anterior cingulate cortex, and constitutes the core mechanism by which PLS2 + genes mediate the improvement of neck pain symptoms.

Meanwhile, PLS2- genes were primarily enriched in GABA-ergic synapse and synaptic membrane-related pathways, serving as another key molecular link for acupuncture to repair the abnormalities of this pain circuit. The balance of excitatory-inhibitory synaptic transmission within the thalamus-ACC circuit is the foundation for maintaining normal pain perception. As the major inhibitory neurotransmitter in the central nervous system, the enhanced function of GABA synapses can effectively suppress the excessive firing of nociceptive neurons in the thalamus and the conduction of nociceptive signals to the anterior cingulate cortex (He et al., 2025). Previous studies have also confirmed that acupuncture exerts analgesic effects by regulating neurotransmitter transmission in the anterior cingulate cortex, particularly through the activation of GABA-ergic neurons in this brain region (Zhu et al., 2024; Wu et al., 2022; Peek et al., 2021). Acupuncture may upregulate the expression of genes related to the formation and function of GABA-ergic synapses within the thalamus-ACC circuit via neurohumoral regulation, repair the impaired inhibitory synaptic transmission function, restore the excitatory-inhibitory signal balance of the circuit, and thereby reduce the abnormal FC between the thalamus and the anterior cingulate cortex to achieve an analgesic effect.

Notably, as the major excitatory neurotransmitter in the central nervous system, glutamate-mediated abnormal hyperactivity of excitatory synaptic transmission constitutes a critical pathological basis for the onset and progression of neck pain. However, no significant enrichment of the glutamatergic pathway was detected in PLS2- genes in this study. This finding may be attributed to the following reasons: the hyperactivity of the glutamatergic pathway is not a primary pathological change in neck pain, but a secondary excitatory imbalance induced by the impaired inhibitory function of the thalamus-ACC circuit. As an exogenous active regulatory intervention, acupuncture exerts its effects not by directly modulating the gene expression of the glutamatergic pathway, but by targeting the repair of the impaired GABA-ergic inhibitory system as the core, antagonizing glutamate-mediated excessive excitatory signal transmission from the downstream, and thus restoring the excitatory-inhibitory balance of the circuit.

The complementary nature of PLS2 + and PLS2- reveals a multi-target integrated mechanism for acupuncture analgesia: peripherally suppressing inflammation (PLS2+) and centrally restoring neural inhibition (PLS2-). By reshaping the “immune-neural interactive homeostasis,” acupuncture both blocks the transmission of peripheral inflammation to the central nervous system and, through neural regulation, inhibits the excessive activation of immune cells. Together, these constitute a complex micro-network for acupuncture analgesia. The improvement of abnormalities within these micro-networks, at the macro-level, “drives” the adaptive remodeling of FC between the thalamus and the ACC, thereby promoting pain recovery.

### The efficacy of acupuncture analgesia

In this study, acupuncture intervention was delivered at cervical tender points in adherence to the classic principle of “taking pain as the acupoint (Yi Tong Wei Shu)” in traditional Chinese medicine (TCM) for the treatment of pain disorders. From a modern pathological perspective, these tender points represent pain-sensitized loci of cervical myofascial injury with local accumulation of inflammatory mediators, which aligns with the abnormal activation of immune-inflammatory pathways identified in the present study. Acupuncture at these sites directly improves local microcirculation and inhibits pain sensitization, forming a local-central synergistic effect with the regulatory modulation of the central thalamocortical pain network. Meanwhile, tender points in this study were quantitatively screened using a pressure algometer to achieve individualized and precise acupoint selection, which avoided the subjectivity inherent in the selection of traditional meridian acupoints, rendered intervention targets more congruent with patients’ specific pain characteristics, and enhanced the precision of acupuncture intervention for mechanical neck pain. This approach thus embodies an integration of classic TCM theory with modern precision pain medicine.

This scientific acupoint selection strategy also laid a foundational basis for acupuncture to exert its specific analgesic effects, which was validated by the clinical outcomes of the present study. Both the VA and SA groups exhibited a significant reduction in VAS scores and improvement in neck pain symptoms after treatment, yet the difference in their effect sizes revealed inherently distinct mechanisms of action. The large effect size in the VA group (*t* = 13.856) reflected the activation of multi-target neurophysiological mechanisms by VA at tender points. In contrast, although the SA group also demonstrated statistically significant improvements post-treatment (*t* = 6.197), its effect size was merely 44.7% of that observed in the VA group. We propose that the analgesic effect of sham acupuncture primarily stems from the placebo effect and the influence of mild sensory stimulation, which also corroborates the role of “non-specific effects” in pain management (Coutaux, 2017). Nevertheless, the intensity and duration of such non-specific effects are far inferior to the multi-scale synergistic regulatory mechanisms mediated by VA. The intergroup difference in therapeutic effects post-treatment further confirmed that the analgesic effect of VA based on tender point selection is independent of the placebo effect and natural disease progression, highlighting the specific biological effects of VA. This not only validates the scientific rationale and clinical efficacy of the adopted acupoint selection strategy but also provides evidence-based medical support and novel clinical insights for acupoint selection in the precision treatment of pain.

However, this study still has several limitations, and targeted follow-up studies will be conducted to address these issues in the future. First, the study adopted a cross-sectional design, with assessments only conducted at baseline and 2 weeks after treatment. The lack of long-term follow-up data at 3, 6, and 12 months post-intervention precludes an elucidation of the long-term maintenance effect of acupuncture analgesia, as well as the dynamic changes in functional connectivity of the thalamocortical network and the expression of related genes. Second, the thalamus defined by the AAL atlas was used as the seed region for FC analysis, which masks the functional specificity of key thalamic nuclei. Third, only left hemisphere gene expression data from the AHBA were utilized in this study, which may have led to the omission of lateralized molecular mechanisms underlying acupuncture analgesia. Fourth, the study only analyzed the correlation between central gene expression and changes in thalamocortical network FC, without detecting peripheral inflammatory factors or conducting correlation analyses between these factors and central gene expression as well as brain functional connectivity. This limitation hinders an in-depth investigation of the regulatory association between peripheral immune-inflammatory responses and central molecular-brain network mechanisms in acupuncture analgesia.

In subsequent research, we will extend the follow-up period to 3, 6, and 12 months, subdivide thalamic nuclei, and adopt gene validation techniques (qPCR and immunohistochemistry) as well as multi-omics integration methods to elucidate the regulatory mechanisms of acupuncture from the peripheral to the central nervous system, thereby providing a more comprehensive theoretical and clinical basis for the precise acupuncture treatment of neck pain.

## Conclusion

The results of this study successfully confirmed our hypothesis that the regional changes in FC before and after acupuncture treatment are indeed spatially correlated with specific transcriptional expression patterns. We found significant changes in FC between the thalamus and the ACC before and after acupuncture treatment, which may be related to acupuncture’s regulation of immune-inflammatory and neuroinhibitory pathways. This work highlights the importance of integrating macroscopic neuroimaging with microscopic expression in understanding acupuncture analgesia.

## Data Availability

The raw data supporting the conclusions of this article will be made available by the authors, without undue reservation.

## Footnotes

1
http://human.brain-map.org

2
https://metascape.org/gp/index.html#/main/step1
